# Investigation of *Citrobacter freundii* clinical isolates in a Chinese hospital during 2020–2022 revealed genomic characterization of an extremely drug-resistant *C. freundii* ST257 clinical strain GMU8049 co-carrying *bla*_NDM-1_ and a novel *bla*_CMY_ variant

**DOI:** 10.1128/spectrum.04254-23

**Published:** 2024-10-10

**Authors:** Mujie Zhang, Zhiqiu Yin, Baozhu Chen, Zhanpeng Yu, Jiaxin Liang, Xiaoyan Tian, Defu Li, Xiaoyan Deng, Liang Peng

**Affiliations:** 1 Department of Clinical Laboratory, Key Laboratory of Biological Targeting Diagnosis, Therapy and Rehabilitation of Guangdong Higher Education Institutes, The Fifth Affiliated Hospital, Guangzhou Medical University, Guangzhou, Guangdong, China; 2 Intensive Care Unit (ICU), The Fifth Affiliated Hospital, Guangzhou Medical University, Guangzhou, Guangdong, China; 3 KingMed School of Laboratory Medicine, Guangzhou Medical University, Guangzhou, Guangdong, China; Post Graduate Institute of Medical Education and Research, Chandigarh, Chandigarh, India

**Keywords:** *Citrobacter freundii*, ST257, ceftazidime-avibactam resistance, NDM-1, CMY variant, IncX3

## Abstract

The emergence of multidrug-resistant *Citrobacter freundii* poses a significant threat to public health. *C. freundii* isolates were collected from clinical patients in a Chinese hospital during 2020–2022. An unusual strain, GMU8049, was not susceptible to any of the antibiotics tested, including the novel β-lactam/β-lactamase inhibitor combination ceftazidime-avibactam. Whole-genome sequencing (WGS) revealed that GMU8049 harbors a circular chromosome belonging to the rare ST257 and an IncX3 resistance plasmid. Genomic analysis revealed the coexistence of two β-lactamase genes, including plasmid-mediated *bla*_NDM-1_ and chromosomal *bla*_CMY_ encoding a novel CMY variant, combined with an outer membrane porin deficiency, which may account for the extreme resistance to β-lactams. Conjugation experiment confirmed that the *bla*_NDM-1_ resistance gene located on pGMU8049 could be successfully transferred to *Escherichia coli* EC600. The novel CMY variant had an amino acid substitution at position 106 (N106S) compared to the closely related CMY-51. Additionally, a GMU8049-specific truncation in an OmpK37 variant that produces a premature stop codon. Moreover, a variety of chromosome-located efflux pump coding genes and virulence-related genes were also identified. Analysis of strain GMU8049 in the context of other *C. freundii* strains reveals an open pan-genome and the presence of mobile genetic elements that can mediate horizontal gene transfer of antimicrobial resistance and virulence genes. Our work provides comprehensive insights into the genetic mechanisms of highly resistant *C. freundii*, highlighting the importance of genomic surveillance of this opportunistic pathogen as a high-risk population for emerging resistance and pathogenicity.

**IMPORTANCE:**

Emerging pathogens exhibiting multi-, extremely, and pan-drug resistance are a major concern for hospitalized patients and the healthcare community due to limited antimicrobial treatment options and the potential for spread. Genomic technologies have enabled clinical surveillance of emerging pathogens and modeling of the evolution and transmission of antimicrobial resistance and virulence. Here, we report the genomic characterization of an extremely drug-resistant ST257 *Citrobacter freundii* clinical isolate. Genomic analysis of GMU8049 with a rare ST type and unusual phenotypes can provide information on how this extremely resistant clinical isolate has evolved, including the acquisition of *bla*_NDM-1_ via the IncX3 plasmid and accumulation through chromosomal mutations leading to a novel CMY variant and deficiency of the outer membrane porin OmpK37. Our work highlights that the emergence of extremely resistant *C. freundii* poses a significant challenge to the treatment of clinical infections. Therefore, great efforts must be made to specifically monitor this opportunistic pathogen.

## INTRODUCTION

Ongoing antimicrobial resistance is a major threat to public health worldwide. Carbapenems are currently used as broad-spectrum, often “last resort” antibiotics, for the treatment of serious bacterial infections. However, since carbapenem use has increased, the continuing emergence of carbapenem-resistant Enterobacteriaceae (CRE) has triggered an unprecedented public health crisis due to limited treatment options. CRE resistance can be induced by intrinsic and acquired mechanisms, including acquired carbapenemases (encoded by e.g., *bla*_NDM_, *bla*_KPC_, *bla*_VIM_, *bla*_OXA_), production of extended spectrum-β-lactamase (ESBL) or AmpC-β-lactamase, mutation of outer membrane proteins (OMPs) porin, alteration of penicillin-binding protein, and efflux pump and biofilm production (1). Among these mechanisms, the rapid spread of acquired carbapenemases via mobile genetic elements (MGEs) contributes significantly to the spread of carbapenem resistance in Enterobacteriaceae (2). According to the degree of cation dependency, carbapenemases are mainly divided into serine enzymes (classes A and D) and metallo-β-lactamases (MBLs) (class B). New Delhi metallo-β-lactamase (NDM), a typical member of class B1 MBLs, confers resistance to all β-lactams except monobactams (3). The NDM coding gene *bla*_NDM_ is usually carried by conjugative plasmids and often coexists with many other resistance determinants, making it one of the most prevalent types of carbapenemases worldwide (4). In particular, the prevalence of the *bla*_NDM_ genes in China has continued to increase in recent years and has attracted increasing attention (5). Ceftazidime-avibactam has been introduced in China as a novel β-lactam/β-lactamase inhibitor combination in the treatment of severe carbapenem-resistant Enterobacteriaceae infections because it is more effective and less toxic than older colistin-based regimens (6). However, in recent years, resistance to ceftazidime-avibactam has been reported among multiple clinical isolates by producing novel variants of the *bla*_CMY_ and *bla*_KPC_ genes (7, 8).

*Citrobacter freundii* is often the causative pathogen of a wide spectrum of nosocomial infections, including respiratory tract, bloodstream, and urinary tract infections (9, 10). It is frequently isolated from the clinical samples of blood, urine, soft tissues, and wounds from patients. In contrast to *Klebsiella pneumoniae* and *Escherichia coli*, *Citrobacter* sp. and in particular *C. freundii*, long considered not to be classical nosocomial pathogens, are rapidly becoming a cause for concern as clinical multidrug-resistant pathogens due to their exceptional ability to accumulate resistance mechanisms (11). The emergence of *C. freundii* carrying carbapenemases coding genes (e.g., *bla*_NDM_, *bla*_KPC_, *bla*_OXA_, *bla*_IMP_) has posed further challenges to patient safety and healthcare systems (12). In addition, *C. freundii* strains were also co-resistant to multiple antibiotics, except for β-lactams, which could further limit the antimicrobial treatment options (13). Due to the phenotypic versatility in colony morphology, as well as in its biochemical, antigenic, and pathogenic behaviors, *C. freundii* is difficult to identify and is often confused with other Enterobacteriaceae pathogens, such as *Salmonella enterica* and *E. coli* (14). The genetic diversity of *C. freundii* is shaped by mechanisms such as homologous recombination, point mutations, and horizontal gene transfer (HGT), which contribute to its phenotypic versatility and antigenic variation (14, 15). This genomic plasticity allows *C. freundii* to rapidly adapt to new niches and challenges, including the development of antimicrobial resistance and the ability to cause infections in humans and animals (14, 15).

In order to control the emergence of *C. freundii* infections and the transmission of antimicrobial resistance, it is important to carry out surveillance and genomic investigations of clinical isolates of this species. In this study, we continuously monitored the *C. freundii* strains in a teaching hospital over a period of 3 years (2020–2022) and then focused on an unusual strain GMU8049, which was not susceptible to any of the antibiotics tested, even including the novel β-lactam/β-lactamase inhibitor combination ceftazidime-avibactam. Whole-genome sequencing (WGS) of antimicrobial-resistant pathogens is increasingly being used to revolutionize antimicrobial resistance surveillance (16, 17). Therefore, the genome of GMU8049 was sequenced to elucidate the genetic mechanisms of this strain. Transfer of a conjugative *bla*_NDM-1_-carrying plasmid was assessed by corresponding conjugation experiment. Our study aims to elucidate the molecular mechanisms of multidrug resistance, pathogenicity, and molecular evolution of clinical *C. freundii* strains, paving the way for the formulation of more effective surveillance and treatment.

## RESULTS AND DISCUSSION

### Overview of *C. freundii* strains isolated from clinical samples

**Fig 1:**
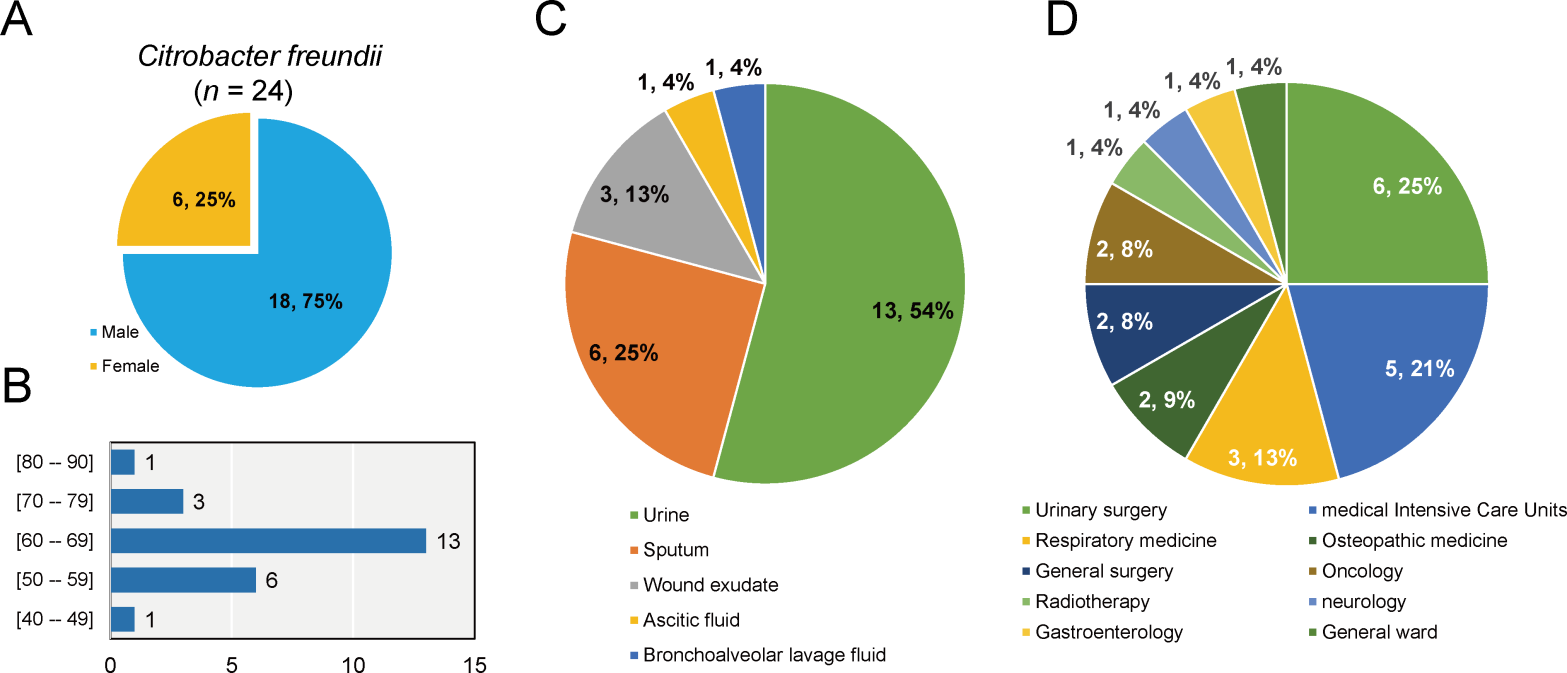
Overview of *Citrobacter freundii* strains (*n* = 24) in the Fifth Affiliated Hospital of Guangzhou Medical University, Guangzhou, Guangdong Province, China, from 2020 to 2022. (A) Proportion of patients carrying *C. freundii* in different gender. (B) Proportion of age range distribution of the patients. (C) Proportion of the types of clinical specimens for *C. freundii*. (D) Proportion of the departments of clinical specimens for *C. freundii*.

From January 2020 to December 2022, a total of 24 clinical cases were identified as *C. freundii* infections. There was no clustering of cases or suspected outbreak during the study period. The number of male patients (*n* = 18; 75.0%) was higher than female patients (*n* = 6; 25.0%) (Fig. 1A). The majority of patients were elderly, aged between 42 and 84 years old. More than half of the cases (*n* = 13; 54.2%) were concentrated in the 60–69 age group (Fig. 1B). Our findings are consistent with a previous study, in which opportunistic *Citrobacter* sp. infections were mainly observed in neonates, the elderly, and immunocompromised persons (18). These *C. freundii* strains were mainly isolated from urine samples (*n* = 13) and sputum (*n* = 6), with a few being isolated from wound exudate samples (*n* = 3), ascitic fluid sample (*n* = 1), and bronchoalveolar lavage fluid sample (*n* = 1). We found that almost half of the strains were isolated from urinary surgery (*n* = 6) and medical intensive care units (ICUs) (*n* = 5). Extensive and unlimited use of routine antibiotics in prophylaxis or treatment of urinary tract infections (UTIs) leads to the emergence of multidrug-resistant uropathogenic bacteria (19). ICU patients usually have longer hospital stays, which increase the risk of nosocomial infections and evolution of multidrug-resistant pathogens (5). Additionally, three strains were also obtained within respiratory medicine unit of the hospital. Empirical antibiotic treatment is commonly used for some respiratory diseases, such as lower respiratory tract infections, and antibiotic-resistant infections are commonly detected in the respiratory department (20).

Antimicrobial susceptibility testing was performed on all 24 *C*. *freundii* strains (Table 1). The results indicated a notable resistance profile among these strains. Specifically, 58.3% demonstrated resistance to ceftriaxone, while 50% were resistant to ceftazidime. Additionally, 33.3% and 29.2% of the strains exhibited resistance to cefepime and cefoperazone-sulbactam, respectively. Aztreonam resistance was observed in 33.3% of the strains, and a relatively high resistance rate of 54.2% was found for piperacillin-tazobactam. Among the carbapenems, resistance was identified in 29.2% of the strains for imipenem, with meropenem and ertapenem showing slightly higher resistance rates of 45.8% and 20.8%, respectively. Regarding non-β-lactams, levofloxacin (fluoroquinolone) exhibited a resistance rate of 58.3%, while sulfamethoxazole (sulfonamide) was resisted by 50.0% of the strains. Furthermore, amikacin, gentamicin, and tigecycline remained within the susceptibility range of 75.0%, 79.2%, and 50.0%, respectively.

**TABLE 1:** Antimicrobial resistance profiles of 24 *C*. *freundii* strains included in this study

Antimicrobial	Class	Antimicrobial resistance rates (%)		
		R*^a^*	I*^a^*	S*^a^*
Aztreonam	β-Lactam	33.3	0.0	66.7
Cefepime	β-Lactam	33.3	0.0	66.7
Ceftazidime	β-Lactam	50.0	4.2	45.8
Cefoperazone-sulbactam	β-Lactam	29.2	4.2	66.7
Ceftriaxone	β-Lactam	58.3	0.0	41.7
Imipenem	β-Lactam	29.2	16.7	54.2
Meropenem	β-Lactam	45.8	0.0	54.2
Ertapenem	β-Lactam	20.8	0.0	79.2
Piperacillin-tazobactam	β-Lactam	54.2	8.3	37.5
Amikacin	Aminoglycoside	0.0	25.0	75.0
Gentamicin	Aminoglycoside	12.5	8.3	79.2
Levofloxacin	Fluoroquinolones	58.3	4.2	37.5
Tigecycline	Tetracycline	4.2	25.0	70.8
Sulfamethoxazole	Sulfonamides	50.0	0.0	50.0

^*a*^ Antimicrobial susceptibility was defined using the CLSI criteria. S, susceptible; I, intermediate; R, resistant.

### Genomic characteristics of an extremely drug resistant *C. freundii* strain GMU8049

Among these clinical isolates, an unusual strain, GMU8049, was not susceptible to any of the antibiotics tested. (Table 2). It was resistant to all examined β-lactams, including aztreonam, cefepime, ceftazidime, cefoperazone-sulbactam, ceftriaxone, imipenem, meropenem, ertapenem, and piperacillin-tazobactam. Furthermore, we observed that GMU8049 was also resistant to the novel β-lactam/β-lactamase inhibitor ceftazidime-avibactam with an inhibition zone diameter of 10 mm using the Kirby-Bauer disk diffusion method. Regarding non-β-lactams, GMU8049 was resistant to gentamicin, levofloxacin, and sulfamethoxazole and was intermediate to amikacin and tigecycline. This strain was isolated from the sputum sample of an elderly male patient aged 64 years old suffering from pneumonia in Guangzhou, on 7 December 2021. WGS and comparative analysis will improve our understanding of the genetic characteristics of multidrug-resistant pathogens (16, 17). Therefore, we report the complete genome and genomic analysis of GMU8049 and elucidate contributing mechanisms leading to the observed phenotypic profile.

**TABLE 2:** Antimicrobial susceptibilities of *C. freundii* GMU8049

Antimicrobial	Class	MIC (mg/L)*^a^*	Inhibition zone diameter (mm)*^b^*	Susceptible*^c^*
Aztreonam	β-Lactam		6	R
Cefepime	β-Lactam	≥32		R
Ceftazidime	β-Lactam	≥64		R
Cefoperazone-sulbactam	β-Lactam	≥64		R
Ceftazidime-avibactam	β-Lactam		10	R
Ceftriaxone	β-Lactam	≥64		R
Imipenem	β-Lactam	≥16		R
Meropenem	β-Lactam		6	R
Ertapenem	β-Lactam	≥8		R
Piperacillin-tazobactam	β-Lactam	≥128		R
Amikacin	Aminoglycoside	32		I
Gentamicin	Aminoglycoside		6	R
Levofloxacin	Fluoroquinolones	≥8		R
Tigecycline	Tetracycline	4		I
Sulfamethoxazole	Sulfonamides	≥320		R

^*a*^ MICs were determined by the VITEK2-Compact drug sensitivity analysis system (bioMérieux, France).

^*b*^ Inhibition zone diameters were determined by the Kirby-Bauer disk diffusion method.

^*c*^ Antimicrobial susceptibility was defined using the CLSI criteria. S, susceptible; I, intermediate; R, resistant.

**Fig 2:**
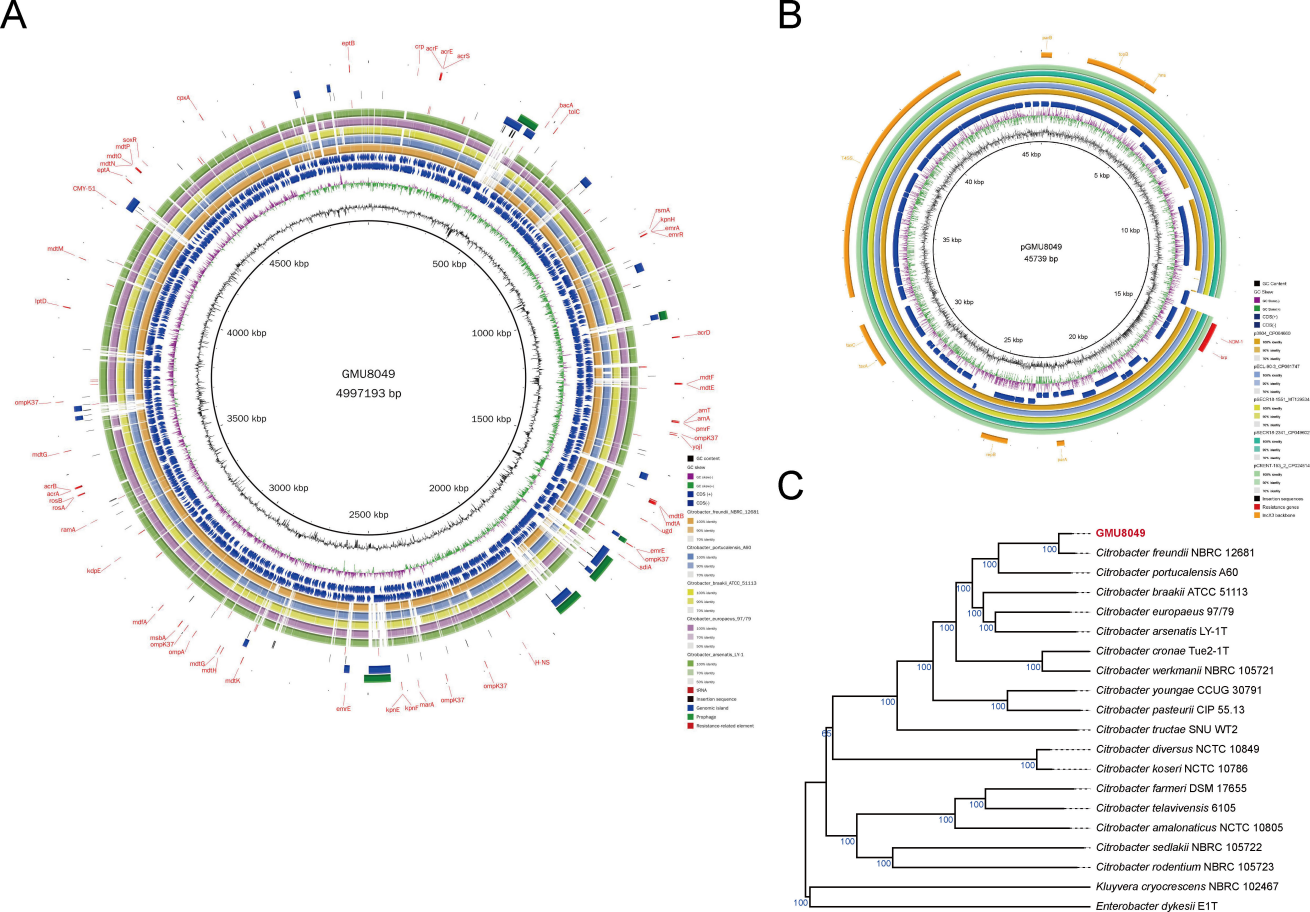
Genomic characteristics of the GMU8049 genome. (A) Circular representation of the GMU8049 chromosome. Rings represent the following features labeled from the inside to the outside: ring 1, GC content; ring 2, GC-skew, green and purple correspond to above- and below-average GC skew, respectively; rings 3–4, blue arrows correspond to plus-strand CDS and minus-stand CDS, respectively; rings 5–9, circular comparison of *C. freundii* NBRC_12681, *C. portucalensis* A60, *C. braakii* ATCC_51113, *C. europaeus* 97/79, and *C. arsenatis* LY-1, respectively; rings 10–14, blocks correspond to potential tRNA loci, ISs, GIs, prophages, and resistance-related elements, respectively. (B) Circular representation of the plasmid pGMU8049. Rings represent the following features labeled from the inside to the outside: ring 1, GC content; ring 2, GC-skew, green and purple correspond to above- and below-average GC skew, respectively; rings 3–4, blue arrows correspond to plus-strand CDS and minus-stand CDS, respectively; rings 5–9, circular comparison of p3804, pECL-90–3, pSECR18-1551, pSECR18-2341, and pCRENT-193_2, respectively; rings 10–12, blocks correspond to potential ISs, resistance genes, and IncX3-type backbone region, respectively. The detail information of each resistance-related genes was listed in Table S4. (C) Genome-based phylogenetic analysis. The phylogenetic tree inferred with genome BLAST distance phylogeny (GBDP) by the Type Strain Genome Server (TYGS) displays the pseudo-bootstrap support values from 100 replications above the branches. The colored boxes to the right of each genome name indicate species, subspecies, GC content, delta statistics, genome size, and protein count, respectively.

The complete genome of GMU8049 consisted of one circular chromosome and one plasmid. It was estimated to have 100.0% completeness with 0.2% contamination. The GMU8049 chromosome was composed of 4,997,193 bp with a GC content of 51.5% (Fig. 2A). The plasmid pGMU8049 was composed of 45,739 bp with 46.5% GC content, constituting 0.9% of the GMU8049 genome (Fig. 2B). A total of 4,959 genes were predicted, of which 4,707 were protein-coding genes, 126 were pseudogenes, and the remaining 126 functional RNA coding genes, including 87 tRNA, 25 mRNA, and 14 non-coding RNA (Table S1). The multilocus sequence typing (MLST) profile of GMU8049 was assigned as ST257 (*arcA*: 5; *aspC*: 51; *clpX*: 53; *dnaG*: 6; *fadD*: 33; *lysP*: 114; *mdh*: 15), which was first isolated from the Netherlands, in 2016. To date, the only representative strain (PubMLST ID: 215) of this ST type is included in the PubMLST database, and no genome of ST257 had been sequenced yet. The complete genome of GMU8049 is the first genome sequence of the *Citrobacter* ST257, thereby increasing our understanding of the genetic characteristics of the rare ST type and relevant antimicrobial resistance.

### Genomic plasticity and phylogenomic analysis of GMU8049

MGEs in bacterial genomes can facilitate DNA acquisition and enable the exchange of genetic material (21, 22). In this study, apart from plasmid pGMU8049, three types of mobile genetic elements (MGEs) were identified in the GMU8049 genome, including genomic islands (GIs), insertion sequences (ISs), and prophages (Table S2). A total of 18 chromosomal GIs were discovered, covering 465.8 kb (9.2%) of the genome size. The GMU8049 chromosome harbored four intact prophages and two incomplete prophages, which together encompassed a region of 305.5 kb and spanned almost 6.1% of the genome. The four complete prophage regions are similar to Salmon_Fels_2 (NC_010463), Salmon_SJ46 (NC_031129), Pseudo_YMC11/07/P54 (NC_030909), and Acinet_vB_AbaS_TRS1 (NC_031098), respectively. These prophages are absent in other closely related *Citrobacter* species (reference genomes) (Fig. 2A), suggesting that the differential distribution of MGEs contributes to strain-level differences. A total of 65 IS elements were identified, of which most ISs (*n* = 61) were located on the chromosome, and four ISs were located on the plasmid pGMU8049.

To achieve the taxonomic inference at the whole-genome level, the phylogenomic tree was constructed using the Type Strain Genome Server (TYGS) (23). As shown in Fig. 2C, strain GMU8049 along with *C. freundii* NBRC 12681^T^ clustered within the genus *Citrobacter*. Pairwise comparisons of genomic properties between GMU8049 and the closely related strains were also conducted using genome BLAST distance on TYGS. The *in silico* DNA-DNA hybridization (DDH) shared between GMU8049 and *C. freundii* NBRC 12681^T^ was 91% (95% confidence interval, 88.8%–92.8%), which was higher than the 70% threshold value for species delimitation (24). Furthermore, a small GC content difference (0.1%) between them was observed (Table S3). Therefore, strain GMU8049 was further confirmed to be a member of the species *C. freundii*.

### Conjugative IncX3 type plasmid pGMU8049 harboring *bla*_NDM-1_

**Fig 3:**
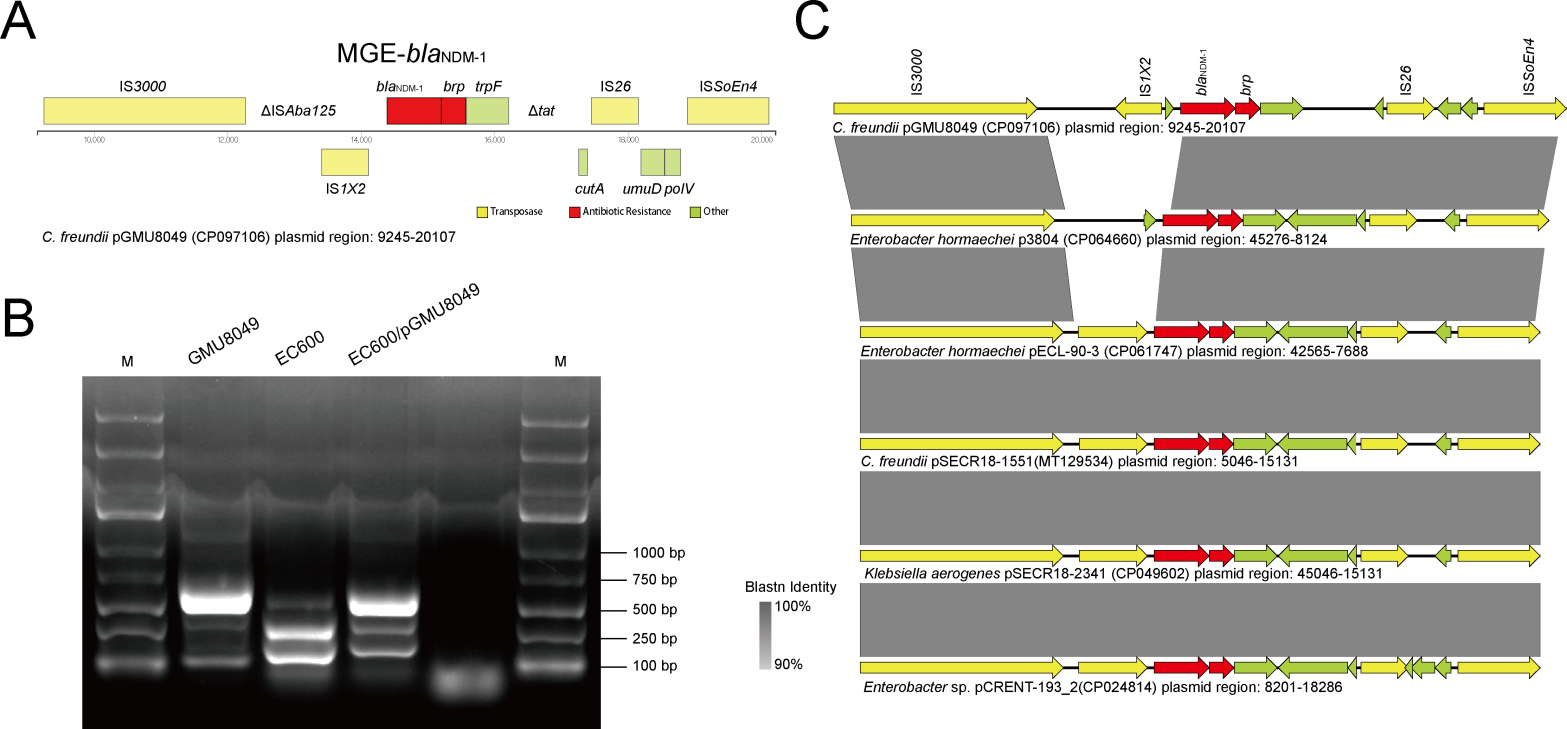
Genetic feature of *bla*_NDM-1_ in the plasmid pGMU8049. (A) Genetic organization of the *bla*_NDM-1_ region in pGMU8049. (B) PCR amplification of *bla*_NDM-1_ gene in the donor strain GMU8049, the recipient strain EC600, and the corresponding transconjugant EC600/pGMU8049. M, molecular weight marker. (C) Comparison of the genetic organization of *bla*_NDM-1_ region. Genes are denoted by arrows. Genes are colored based on their functional classification. Gray shading indicates homologous regions.

We found that plasmid pGMU8049 carried an NDM enzyme coding gene, *bla*_NDM-1_ (M3L74_00080). This carbapenemase-coding gene has posed a serious threat to public health and is commonly inserted into transferable plasmids, which facilitates its rapid spread among various species of Enterobacterales (25). The genetic organization of the *bla*_NDM-1_ region in pGMU8049 was similar but not identical to other plasmid-based *bla*_NDM_ regions in *C. freundii* (9). As shown in Fig. 3A, a *brp*_MBL_ gene (M3L74_00085) was present downstream of *bla*_NDM-1_, with both genes being separated by 3 bp. The *brp*_MBL_ gene encodes a bleomycin resistance protein, which is considered to stabilize the plasmid-borne *bla*_NDM-1_ gene (26). *bla*_NDM-1_ and *brp*_MBL_ were flanked upstream by IS*3000*-ΔIS*Aba125*-IS1 × 2 and downstream by *trpF*-Δ*tat-cutA*-IS*26-umuD-polV*-IS*SoEn4*. These ISs surrounding resistance genes play an important role in the horizontal transmission of antimicrobial resistance. The pGMU8049 plasmid belonged to the incompatibility group IncX3 according to PlasmidFinder 2.0.1 (27). IncX3 plasmids, as one of the major vehicles for *bla*_NDM_ genes, have reportedly played a significant role in the spread of carbapenem resistance across Asia, particularly in China (28, 29). pGMU8049 had a typical IncX3 backbone, including replication initiation (IncX3 type *repB*), segregation (*parA* and *parB*), maintenance (*hns* and *topB*), conjugal type IV secretion system (*pilX1* to *pilX11*), and DNA transfer protein (*taxA* and *taxC*) (Fig. 2B). The *bla*_NDM_-carrying IncX3 plasmids have the ability to spread widely among different Enterobacterial species at a wide range of temperatures (30). In this study, a conjugation experiment was performed to evaluate the potential of pGMU8049 to be transferred to the rifampicin-resistant *E. coli* strain EC600. We observed that the plasmid of GMU8049 was successfully transferred to the recipient EC600. The obtained transconjugant strain, designated EC600/pGMU8049, exhibited an imipenem-resistant phenotype (Fig. S1). PCR and sequencing analysis further confirmed that the GMU8049 had transferred *bla*_NDM-1_ to EC600 (Fig. 3B). Our results confirmed the efficient transfer of *bla*_NDM-1_ via the IncX3 plasmid pGMU8049.

BLASTn analysis against the National Center of Biotechnology Information (NCBI) Nucleotide collection (nt) database showed that pGMU8049 shared >99.9% nucleotide identity and >95% coverage with the *bla*_NDM-1_-carrying plasmid p3804 (CP064660; isolated from Hangzhou, China in 2019) and pECL-90-3 (CP061747; isolated from Nanjing, China in 2017) from *Enterobacter hormaechei*, pSECR18-1551 (MT129534; isolated from South Korea in 2018) from *C. freundii*, pSECR18-2341 (CP049602; isolated from South Korea in 2018) from *Klebsiella aerogenes*, and pCRENT-193_2 (CP024814; isolated from South Korea in 2013) from *Enterobacter* sp. (31) (Fig. 3C). The homologous *bla*_NDM-1_ region in these plasmids exhibited similar organizations of gene loci and presented identities of nearly 100% between nucleotide sequences. The fact that these similar plasmids with high homologies have been found in different species of the Enterobacteriaceae in various East Asian countries and regions further highlights that IncX3 plasmids have emerged as a common mobile element mediating interspecies/intraspecies horizontal transfer of *bla*_NDM-1_ in East Asia, particularly in China. The only difference is that the *bla*_NDM-1_ region in pGMU8049 had undergone an insertion of IS*1 × 2* and the pseudogenization of *tat*. Moreover, pGMU8049 possessed a pseudogenized IS*Aba125*, which was also found in *E. hormaechei* p3804 (Fig. 3C).

### A novel *bla*_CMY_ variant, one outer member porin mutation, and diverse efflux pump coding genes in the GMU8049 chromosome

**Fig 4:**
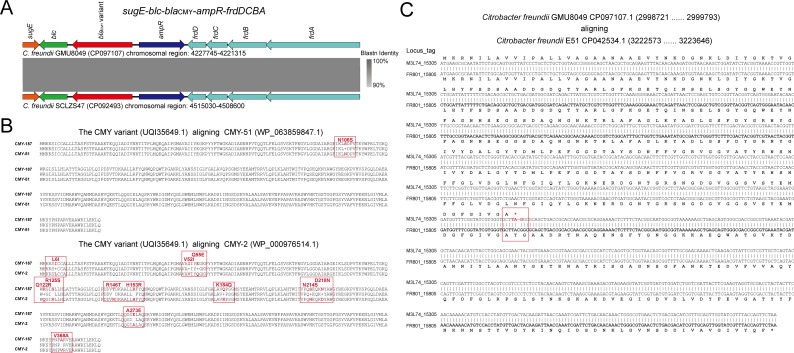
Genetic feature of novel CMY variant and OmpK37 in GMU8049. (A) Comparison of the genetic organization of *bla*_CMY_ region in GMU8049 chromosome. Genes are denoted by arrows. Genes are colored based on their functional classification. Gray shading indicates homologous regions. (B) Alignments of amino acid sequence of the CMY variant, CMY-51, and CMY-2. The sequence in the red box is the mutated region. (C) Alignment of nucleotide sequences of the *ompK37* mutation (M3L74_15305) and its the most homologous counterpart (FR801_15805). *, Stop codon.

In addition to plasmid-mediated *bla*_NDM-1_, the GMU8049 chromosome also possessed an AmpC β-lactamase coding gene *bla*_CMY_ (M3L74_21105). Similar to other intrinsic *bla*_CMY_ genes found in *C. freundii* (32), *bla*_CMY_ (M3L74_21105) and its regulator gene *ampR* (M3L74_21100) in the GMU8049 chromosome were flanked downstream by the *blc* gene (encoding an outer membrane lipocalin) and the *sugE* gene (encoding quaternary ammonium compound efflux SMR transporter), and upstream by the *frdABCD* operon (encoding a fumarate reductase) (Fig. 4A). AmpC β-lactamases are the dominant causes of resistance to broad-spectrum cephalosporins in Enterobacteriaceae (33). For GMU8049, this chromosomal *bla*_CMY_ encoded a novel CMY-2 type β-lactamase variant. The protein sequence of CMY variant (UQI35649.1) possessed one amino acid substitution at position 106 (N106S) relative to the most homologous counterpart CMY-51 (WP_063859847.1) (Fig. 4B). When compared to classical CMY-2 (WP_000976514.1), the CMY variant enzyme possessed 12 amino acid substitutions, including L6I, V52I, Q55E, Q122R, R125S, R146T, H153R, K184Q, N214S, D218N, A273E, and V368A (Fig. 4B). The *bla*_CMY_ genes are evolving to hydrolyze broad-spectrum cephalosporins more efficiently (34). Recently, several CMY variants, CMY-172, CMY-178, and CMY-185, have been reported to confer high-level resistance to ceftazidime-avibactam in clinical isolates, particularly in China (7, 8). As a novel β-lactam/β-lactamase inhibitor, the combination drug ceftazidime-avibactam has powerful activity against clinical isolates of Enterobacterales producing ESBL or AmpC, including CMY-type cephalosporinase (35). It has been marketed in China since September 2019 for the treatment of carbapenem-resistant Enterobacterales infections (6). In this study, GMU8049 displayed ceftazidime-avibactam resistance (inhibition zone diameter = 10 mm) (Table 2). It can be inferred that the novel *bla*_CMY_ variant may confer resistance to broad-spectrum cephalosporins, especially for ceftazidime-avibactam. The potential biological function of this variant in ceftazidime-avibactam resistance and relevant evolutionary dynamics are important foci in our further study to enable us to fully understand the emergence of resistant mutants in clinical isolates.

In addition to acquired resistance mechanisms, carbapenem resistance can also result from intrinsic mechanisms via mutations in OMP proteins, which do not allow penetration by certain β-lactams (36). The mutation in OmpK families has been reported to be associated with carbapenem nonsusceptibility, likely resulting from reduced outer membrane permeability (37). Genomic analysis identified six chromosomal genes encoding potential OmpK37 variants. Among them, one *ompK37* variant (M3L74_15305) has pseudogenized due to a premature stop codon via the loss of base (C) at position 627 (Fig. 4C), which leads to the truncation in OmpK37 previously associated with reduced outer membrane permeability (38). BLASTn analysis against the NCBI nt database revealed that this OmpK37 coding gene was conserved in *C. freundii* and specifically pseudogenized in the GMU8049 genome. Overall, the presence of two β-lactamases coding genes (*bla*_NDM-1_ and *bla*_CMY_ variant) and a porin mutation in the GMU8049 genome seemed to account for its displayed phenotypic resistance to all tested β-lactams, including ceftazidime-avibactam.

Additionally, the GMU8049 chromosome was equipped with a variety of efflux pump coding genes (Table S4) (39). Most *bla*_NDM-1_-positive *C. freundii* isolates are co-resistant to multiple antibiotics. Indeed, GMU8049 was also nonsusceptible to multiple non-β-lactam antibiotics (Table 2). Therefore, these efflux pump coding genes also play an important role in its extremely drug resistance, and in combination with resistance genes, contribute to the resistome of GMU8049.

### Virulence-related genetic profile in GMU8049

**Fig 5:**
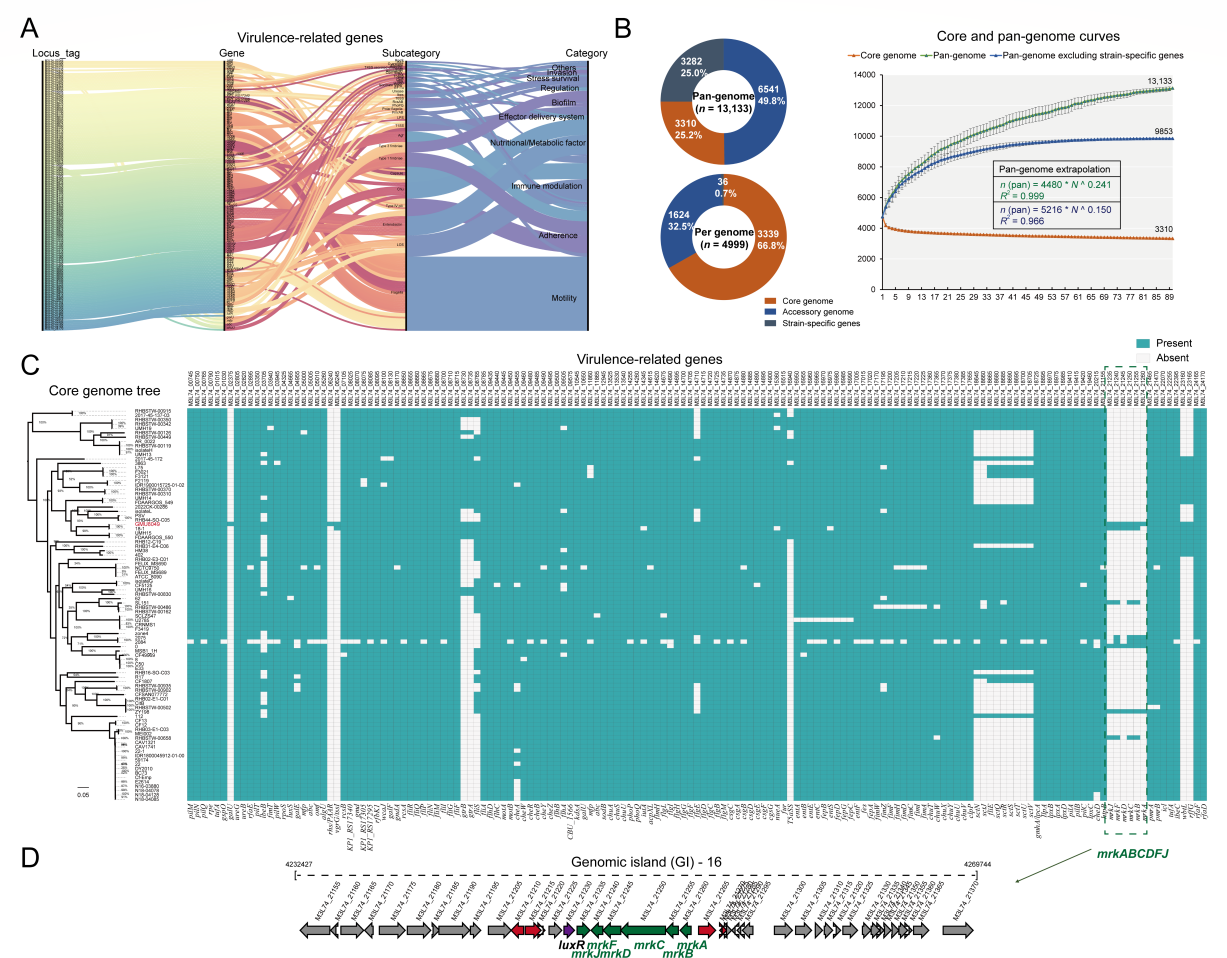
Genetic features of virulence-related genes in GMU8049 and pan-genome analysis. (A) Sankey diagram showing the associations between virulence-related genes in GMU8049, virulence genes, and subcategory and category of corresponding virulence factors. The detail information of each virulence-related genes was listed in Table S5. (B) Pie chart represents the percentages of the core, accessory, and strain-specific gene families participating in the pan-genome (upper) and per genome (lower). Progressive curves for the core genome, pan-genome, and pan-genome excluding strain-specific genes were estimated for the 90 *C*. *freundii* genomes. The curves exhibit a downward trend for the core gene families and an upward trend for the pan-gene families with the addition of genomes. Deduced mathematical functions of the pan-genome curves are shown in the graph. (C) Heatmap of the distribution of virulence-related genes in *C. freundii*. Single-nucleotide polymorphisms (SNPs) across 2,998 single-copy core gene families shared by 90 *C*. *freundii* genomes were employed to generate the core genome tree based on the maximum likelihood (ML) method. Next to the tree, color blocks and white blocks represent the presence and absence of virulence-related genes, respectively. The genome order is based on the core genome tree. (D) Genetic organization of the GI-16 gene cluster. The *mrkABCDFJ* genes are indicated in green. Adjacent genes encoding putative transposable elements (red) and transcriptional regulator are indicated in red and purple, respectively.

To provide a comprehensive view of the pathogenic potential of GMU8049, we investigated the virulence-related genetic profile. A total of 153 genes carried by strain GMU8049 were found to match with known virulence genes (Table S5), which were mainly associated with “Motility” (*n* = 45), “Adherence” (*n* = 30), “Immune modulation” (*n* = 26), and “Nutritional/Metabolic factor” (*n* = 22) (Fig. 5A). The gene loci of macromolecular secretion systems included those associated with two type I secretion systems (T1SSs), one type IV pilus (T4P), one type V secretion system (T5SS), and flagellum secretion system. T1SS can secrete many proteins, including exotoxins involved in pathogenesis, bacteriocins involved in antibacterial activity, and extracellular proteases involved in nutrient acquisition (40). The T4P gene cluster was identified to be required for colonization of the gastrointestinal tract by *Citrobacter rodentium* (41). The expression of the flagellar system in *C. freundii* has been reported to enhance motility, thereby contributing to adherence to host cells and inducing cytotoxicity (42). As the classical autotransporter, T5aSSs can function as enzymes, adhesins, cytotoxins, or mediate bacterial motility (43). The GMU8049 genome also harbored multiple fimbrial operons, including the *csg* operon encoding curli fimbriae, the *fim* operon encoding type 1 fimbriae, and the *mrk* operon encoding type 3 fimbriae. These fimbrial operons mediate fimbrial adhesin and biofilm formation, thereby contributing to virulence (44, 45). The majority of remaining virulence-related genes included a lipopolysaccharide (LPS) biosynthetic operon with the function of adhesion and endotoxin, an *ent* operon involved in the biosynthesis of siderophore enterobactin, and a *chu* operon encoding haem iron-transport system responsible for heme uptake. The occurrence of diverse virulence-related genes underlies the potential pathogenesis of GMU8049. Indeed, as an opportunistic pathogen, *C. freundii* can colonize the human gastrointestinal tract and induce nosocomial respiratory, urinary tract, and bloodstream infections in susceptible patients (46).

### Pan-genome analysis reveals potential genetic innovation of *C. freundii*

Pathogenic strains emerge largely due to genetic innovation regarding acquisition of virulence and antimicrobial resistance genes via HGT. Pan-genome analysis constitutes a powerful method for understanding the genetic potential of a bacterial species (47). To construct the *C. freundii* pan-genome, all available complete genomes (*n* = 90, including GMU8049) of this species defined by the NCBI GenBank database were collected (Table S6). A total of 13,133 homologous gene families were identified (Fig. 5B). Among these, 3,310 (25.2%) are present across all genomes, representing the core genome; 6,541 (49.8%) which comprise the largest group representing the accessory genome, are present in at least one *C. freundii* genome; the remaining 2,429 (21.0%) are only present in one genome representing the strain-specific gene content (Fig. 5B). On average, one genome is composed of 3,339.4 ± 13.5 core genes, 1,623.8 ± 275.6 accessory genes, and 36.5 ± 77.3 strain-specific genes. The accessory genes of *C. freundii* is abundance, contributing to the genetic diversity of the species. The accumulation curve for each genome added was fit to Heaps’ power law function (*n* = ${кN}^{\gamma }$) to determine whether the pan-genome is open (*γ* ≥ 0; a constantly increasing collection of distinct genes) or closed (*γ* < 0). An open pan-genome suggests that a species has a high capacity for the exchange of genetic elements, whereas a closed pan-genome indicates a limited capacity for the acquisition of foreign genes (48). The inferred growth exponent (*γ*) value of 0.241 suggests that the pan-genome of *C. freundii* is open (Fig. 5B), indicating that this species has an gene pool that enables it to continuously acquire exogenous genetic elements, such as antimicrobial resistance genes and virulence-related genes. Additionally, when excluding strain-specific genes, a plateau appears in the pan-genome accumulation curve. The core genome curve shows that the core genome size decreases with the addition of genomes (Fig. 5B).

We performed a phylogenetic analysis based on single nucleotide polymorphisms (SNPs) across 2,998 single-copy core gene families. As shown in Fig. 5C, GMU8049 along with strain 18–1 formed a monophyletic clade, which was deeply nested within the tree. The distribution of the virulence-related genes carried by GMU8049 among other *C. freundii* genomes was also investigated (Fig. 5C). Most of these identified virulence-related genes were also present in other *C. freundii* genomes. Some of them were scattered, which may be attributed to HGT. For example, the complete type 3 fimbriae biosynthesis operon (*mrkABCDFJ*) present in only three genomes (including GMU8049) was located in the genomic island region (designated GI-16) of the GMU8049 chromosome and was surrounded by transposable elements (Fig. 5D), indicating the occurrence of HGT. Type 3 fimbriae has been reported to contribute to nosocomial infection by *K. pneumoniae* as appendages mediating biofilm formation on biotic and abiotic surfaces (49). Previous studies revealed the transmission of the *mrkABCDFJ* operon between Enterobacteriaceae pathogens via HGT (50). We observed that the *mrkABCDFJ* operon in GMU8049 was highly homologous to that of *K. pneumoniae* Z0117KP0003, with 91.4%–99.5% identities of protein sequences in the operons. Meanwhile, two homologous operons also exhibited identical organizations of gene clusters (Fig. S2). Taken together, these data indicated that GMU8049 acquired the *mrkABCDFJ* operon via HGT. Our result further supports evidence that *C. freundii* members may enhance pathogenicity by acquiring exogenous virulence-related genes, thereby posing potential risks for emerging infections.

### Conclusions

In this study, we analyzed 24 *C*. *freundii* clinical isolates from 2020 to 2022 in the Fifth Affiliated Hospital of Guangzhou Medical University, Guangdong Province, China. Among them, we identified and sequenced an unusual ST257 strain, GMU8049, which was not susceptible to any of the antibiotics tested, including ceftazidime-avibactam. The GMU8049 genome harbored a circular chromosome belonging to ST257 and an IncX3 type plasmid pGMU8049. The genome of GMU8049 contains tRNA loci and diverse MGEs, including prophages, GIs, ISs, and plasmid. The IncX3 plasmid, pGMU8049, co-carried *bla*_NDM-1_ and *brp*_MBL_. The *bla*_NDM-1_ region was similar to other plasmid-based *bla*_NDM_ regions in *Enterobacteriaceae*, except for an IS*1 ×* 2 insertion and the pseudogenization of the *tat* gene. Conjugation experiment reveals that GMU8049 successfully transferred *bla*_NDM-1_ and carbapenem nonsusceptible phenotype to the recipient strain *E. coli* EC600, which confirmed the horizontal transfer characteristic of NDM-1. A novel CMY-2 AmpC β-lactamase variant was found on the chromosome, which had a similar backbone appearing in other *C. freundii*. When compared to the closely related CMY-51, the CMY variant has a substitution (N106S) of the consecutive amino acid at position 106. Considering other CMY variants recently reported (7, 8, 34), we inferred that this variant might confer high-level resistance to ceftazidime-avibactam in GMU8049. Furthermore, in the GMU8049 chromosome, a strain-specific *ompK37* mutant (M3L74_15305) had a C base deletion at position 627, leading to a frame shift and premature stop codon. This mutation might lead to the truncation of the outer membrane porin OmpK37, associated with reduced outer membrane permeability for carbapenems. Moreover, we also identified a variety of efflux pump coding genes located on the GMU8049 chromosome, which might be associated with observed nonsusceptibility to non-β-lactams, including gentamicin, levofloxacin, tigecycline, and sulfamethoxazole. GMU8049 also harbored a variety of virulence-related elements, indicating its pathogenic potential.

As the first genome sequence of *C. freundii* ST257, our work provides fresh insights into the genomic diversity of this species and potential evolutionary mechanisms of antimicrobial resistance of this clinical isolate. An open pan-genome and the presence of MGEs carrying antimicrobial resistance and virulence genes in *C. freundii* poses a risk to public health. We highlight the emergence of extremely resistant *C. freundii* is of major concern for patient care and public health, therefore continuous surveillance and monitoring of the dissemination of antimicrobial resistance of this opportunistic pathogen are necessary.

## MATERIALS AND METHODS

### Bacterial strains and antimicrobial susceptibility testing

Twenty-four *C. freundii* strains were isolated from 18 male and 6 female patients in the Fifth Affiliated Hospital of Guangzhou Medical University, Guangzhou, Guangdong Province, China, between 2020 and 2022. The sample collection and isolate identification were performed by the Department of Clinical Laboratory of the hospital. Clinical strains were first identified by MALDI-TOF mass spectrometry (MS) (Bruker, Germany). All strains were cultivated in Luria-Bertani broth at 37°C for 12 h and on tryptic soy agar plates at 37°C for 24 h. Single colonies were inoculated into the LB medium and cultivated at 37°C for 18 h. Antimicrobial susceptibility testing of these strains was performed by the VITEK2-Compact drug sensitivity analysis system (bioMérieux, France) and/or the Kirby-Bauer disk diffusion method according to standard protocols. The interpretative criteria were from the Clinical and Laboratory Standards Institute (CLSI) document M100 (51).

### Genome sequencing and annotation of a novel strain GMU8049

A novel strain GMU8049 was isolated from the sputum sample of an elderly male patient in Guangzhou, China’s Guangdong Province, on 7 December 2021. This 64-year-old man was diagnosed with severe pneumonia. The genomic DNA of strain GMU8049 was extracted using HiPure Bacterial DNA Kits (Magen, Guangzhou, China) and was then sequenced using the PacBio Sequel platform and Illumina NovaSeq PE150 with 2 × 150 bp paired-end reads. *De novo* genomes were assembled using Falcon v0.3.0 (52). The genome data of GMU8049 was deposited and annotated in NCBI RefSeq database (accession number CP097107: chromosome; CP097106: plasmid). The quality of the genome was assessed using CheckM v1.0.13 (53). TYGS was used for accurate genome-based taxonomy of GMU8049 (23).

### Comparative genomic analysis

The features of the genome and comparisons thereof were performed using the BLAST Ring Image Generator (BRIG) (54). The online interface of IslandViewer 4 (55) was utilized to identify GIs. ISs were predicted using the online interface of ISfinder (56). The prophages were predicted using the online interface of PHAge search tool—Enhanced Release (PHASTER) (57). TYGS was used for accurate genome-based taxonomy (23). The *in silico* DDH values were calculated using the genome-to genome distance calculator 2.1 (GGDC) (24). The MLST profile of GMU8049 was implemented at the online interface of PubMLST (https://pubmlst.org/organisms/citrobacter-spp). A BLASTn search of the NCBI nt database was conducted using pGMU8049 sequence as the query sequence. Plasmid replicon was identified by using the PlasmidFinder database. The pseudogenization of genes was detected using the NCBI Prokaryotic Genome Annotation Pipeline (PGAP) v6.1 (58). Antimicrobial resistance genes were detected using Abricate v1.0.1 with Comprehensive Antibiotic Resistance Database (CARD) database (39). To identify the virulence factors, the protein sequences of GMU8049 were aligned using BLASTp with an *E*-value cutoff <1*e−*6, an identity >60%, and a coverage >60% against the data set (VFDB_setA_2023) from the Virulence Factor Database (VFDB) (59). The detection of macromolecular systems were performed using the TXSScan v1.1.1 module (60).

### Pan-genome analysis

Orthologous groups of protein families of pan-genome were delimited using OrthoFinder2 software with DIAMOND method (61, 62). The OrthoFinder output files (Orthogroup_Sequences folder) were used to extract pan-genome families (the totality of all genes found across strains), core genome families (genes shared among all strains), accessory genome families (genes shared among more than one strain, but not in all), and strain-specific genes (genes found only in one strain). Curve fitting of the pan-genome was performed using a power-law regression based on Heap’s law (*n* =${кN}^{\gamma }$) (63, 64), where *N* is the number of genomes, *κ* is a proportionality constant, the growth exponent *γ* > 0 indicating an open pan-genome. A descriptive statistical analysis was generated using OriginPro 9 software with Allometric1 model.

### Phylogenetic analysis

The core genome phylogenetic analysis was performed based on single nucleotide polymorphisms (SNPs) across single-copy core gene families extracted from the OrthoFinder output files (Single_Copy_Orthologue_Sequences folder). Nucleotide sequences of the single-copy core gene families (*n* = 2,998) were extracted according to the protein accession numbers and then aligned using the MAFFT v7.508 software (65). The set of SNPs presented in single-copy core gene families was extracted and then integrated according to the arrangement of the genes on the GMU8049 genome (complete genome). To avoid phylogenetic confusion, we identified and removed the putative recombinational regions from the SNPs set using ClonalFrameML v1.12 software (66). The maximum likelihood (ML) tree was constructed using MEGA 7 (67) with the general time reversible (GTR) model and 100 bootstrap replicates.

### Plasmid conjugation experiment

The co-transfer of *bla*_NDM_-carrying IncX3 plasmid was investigated by filter mating using *C. freundii* GMU8049 as the donor and *E. coli* EC600 (rifampicin resistant) as the recipient. Both the donor GMU8049 and the recipient EC600 were grown to logarithmic phase (OD_600_ of approximately 0.6) at 37°C at 220 rpm in 5 mL of fresh LB broth. Then, 1 mL cultures of the donor cells and 1 mL cultures of the recipient cells were mixed and inoculated into 8 mL Mueller-Hinton (MH) broth at 37°C overnight. The 100 µL mixture was inoculated onto the selective medium (MH agar plates containing 4 µg/mL imipenem and 256 µg/mL rifampicin). After incubation at 37°C overnight, transconjugants that grew on the selective medium were collected and identified using MALDI-TOF MS. The presence of the *bla*_NMD-1_ gene in the positive transconjugant strain, designated EC600/pGMU8049, was investigated using Polymerase chain reaction (PCR) with the primer pairs NDM1-F (5′- CAGTCGCTTCCAACGGTTTG-3′) and NDM1-R (5′- ATCACGATCATGCTGGCCTT-3′) designed by Primer Premier v6.0. The PCR-amplified product was confirmed using gel electrophoresis and Sanger sequencing. The resulting sequence was 502 bp and showed 100% nucleotide identity with the corresponding sequence of the donor gene *bla*_NMD-1_ (M3L74_00080).

## Data Availability

Complete sequence of the GMU8049 genome was deposited in NCBI GenBank database (accession number: chromosome: CP097107; plasmid: CP097106).

## SUPPLEMENTAL MATERIAL

Supplemental figures spectrum.04254-23-s0001.docx Fig. S1 and S2.

Supplemental tables spectrum.04254-23-s0002.xlsx Tables S1 to S6.

ASM does not own the copyrights to Supplemental Material that may be linked to, or accessed through, an article. The authors have granted ASM a non-exclusive, world-wide license to publish the Supplemental Material files. Please contact the corresponding author directly for reuse.
