# Supplementary material for: Investigation of Citrobacter freundii clinical isolates in a Chinese hospital during 2020–2022 revealed genomic characterization of an extremely drug-resistant C. freundii ST257 clinical strain GMU8049 co-carrying blaNDM-1 and a novel blaCMY variant
Source: Microbiol Spectr. 2024 Oct 10;12(11):e04254-23. doi: 10.1128/spectrum.04254-23 (PMC11537026; doi:10.1128/spectrum.04254-23)
Supplement: Supplemental figures — Fig. S1 and S2. [file spectrum.04254-23-s0001.docx]

**SUPPLEMENTAL MATERIAL FIGURE**


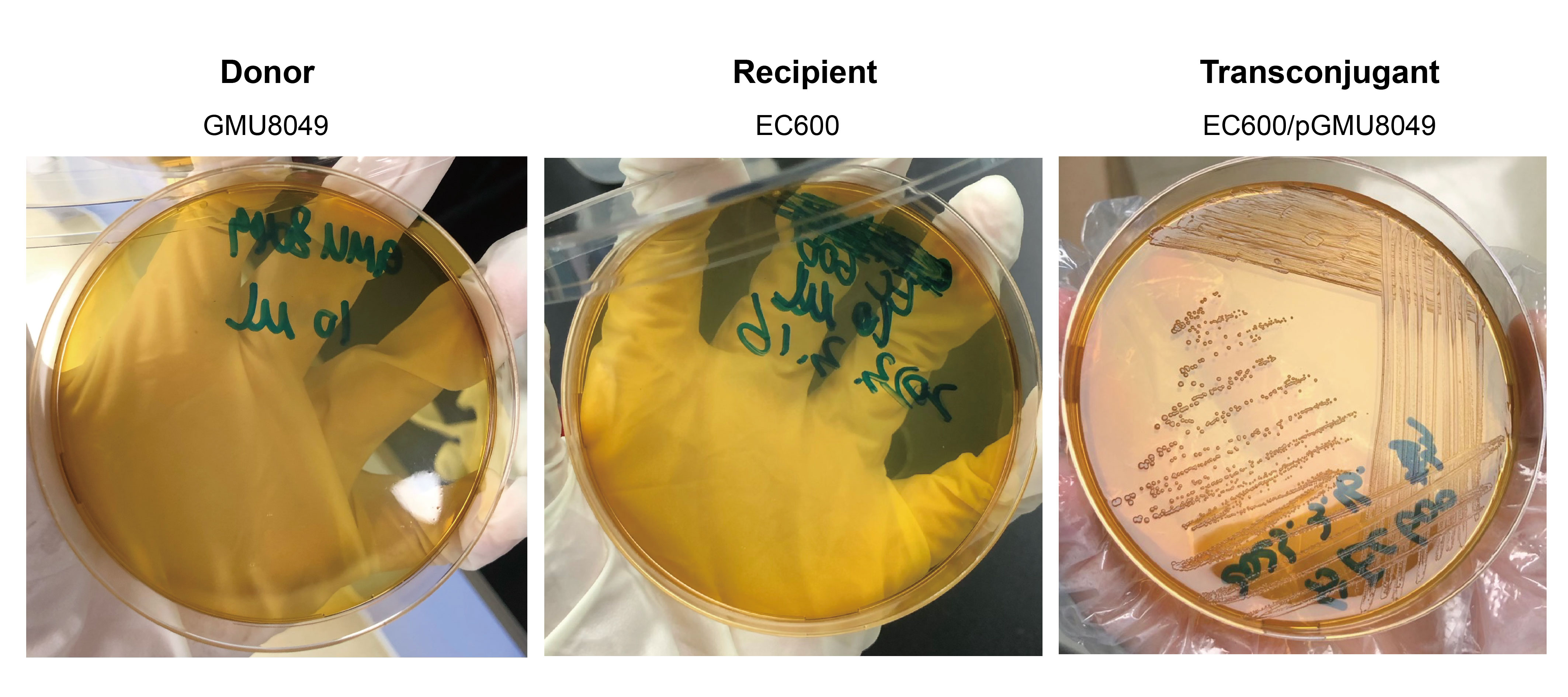


**FIG S1.** The growth of bacteria for plasmid conjugation experiment on MH agar plates containing both imipenem and rifampicin antibiotics. Donor and recipient cells were GMU8049 and EC600, respectively. The corresponding transconjugants (EC600/pGMU8049) grow on plates containing both imipenem and rifampicin, indicating successful conjugation of *bla*_NDM-1_.


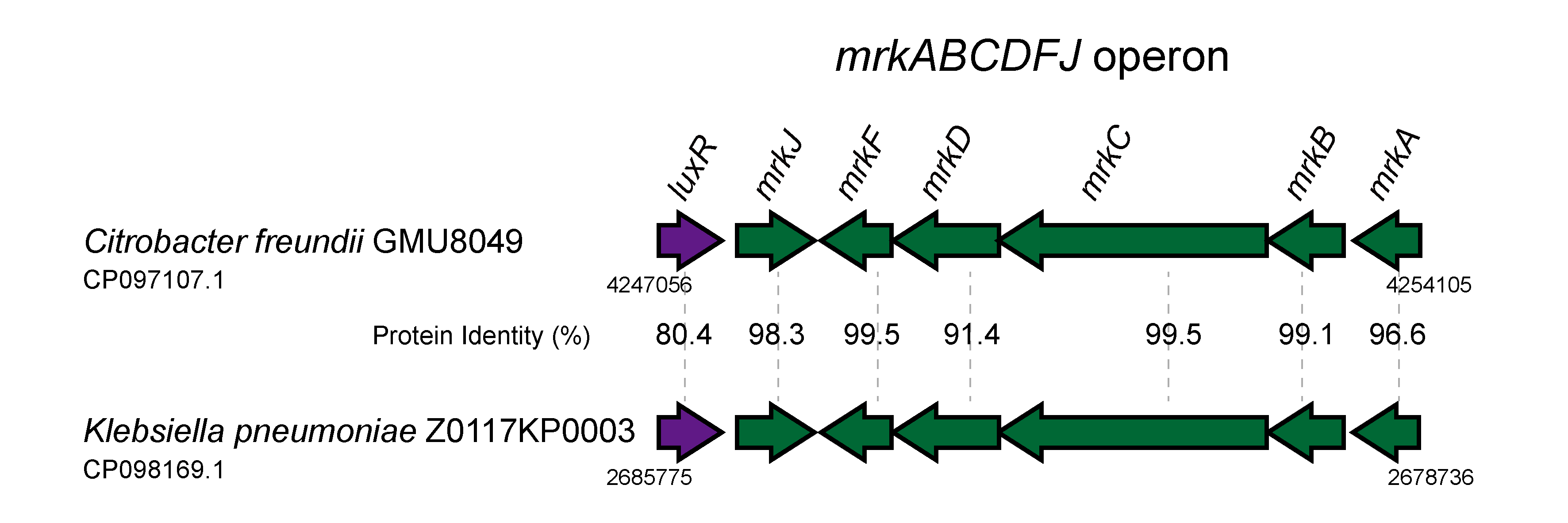


**FIG S2.** Genetic organization of the *mrkABCDFJ* operon in GMU8049 and *Klebsiella pneumoniae* Z0117KP0003 (CP098169.1) and the comparison of the protein sequences between two homologous operons. The amino acid identities for each pair of the homologous proteins are shown.
